# Outlier-resilient complexity analysis of heartbeat dynamics

**DOI:** 10.1038/srep08836

**Published:** 2015-03-06

**Authors:** Men-Tzung Lo, Yi-Chung Chang, Chen Lin, Hsu-Wen Vincent Young, Yen-Hung Lin, Yi-Lwun Ho, Chung-Kang Peng, Kun Hu

**Affiliations:** 1 Research Center for Adaptive Data Analysis & Center for Dynamical Biomarkers and Translational Medicicne, National Central University, Taoyuan, Taiwan; 2 Medical Biodynamics Program, Division of Sleep and Circadian Disorders, Brigham and Women's Hospital, 221 Longwood Avenue, Boston, MA 02115, USA; 3 Graduate Institute of Communication Engineering, National Taiwan University, Taipei, Taiwan; 4 Department of Internal Medicine, National Taiwan University Hospital and National Taiwan University College of Medicine, Taipei, Taiwan; 5 Division of Interdisciplinary Medicine and Biotechnology, Beth Israel Deaconess Medical Center/Harvard Medical School, Boston, Massachusetts, USA; 6 Division of Sleep Medicine, Harvard Medical School, Boston, MA, USA

## Abstract

Complexity in physiological outputs is believed to be a hallmark of healthy physiological control. How to accurately quantify the degree of complexity in physiological signals with outliers remains a major barrier for translating this novel concept of nonlinear dynamic theory to clinical practice. Here we propose a new approach to estimate the complexity in a signal by analyzing the irregularity of the sign time series of its coarse-grained time series at different time scales. Using surrogate data, we show that the method can reliably assess the complexity in noisy data while being highly resilient to outliers. We further apply this method to the analysis of human heartbeat recordings. Without removing any outliers due to ectopic beats, the method is able to detect a degradation of cardiac control in patients with congestive heart failure and a more degradation in critically ill patients whose life continuation relies on extracorporeal membrane oxygenator (ECMO). Moreover, the derived complexity measures can predict the mortality of ECMO patients. These results indicate that the proposed method may serve as a promising tool for monitoring cardiac function of patients in clinical settings.

Many physiological variables such as motor activity and heart rate display seemingly irregular fluctuations over a wide range of time scales^1^,^2^. Under normal healthy conditions, these physiological fluctuations are neither random nor too regular, possessing robust, multi-scale dynamic patterns that are independent of external influences^3^,^4^,^5^. Such a complexity in physiological fluctuations has been accepted as a hallmark of healthy physiology and is believed to reflect system adaptability in response to constant changes of internal and external inputs. Numerous studies have supported this theory of complexity by showing that physiological fluctuations become either too random or too regular with aging and under pathological breakdowns^6^,^7^,^8^,^9^,^10^.

Despite the physiological importance of the complexity theory, its application to clinical studies has been hindered by the lack of algorithms that can be easily implemented for accurate estimation of the degree of complexity in physiological fluctuations^3^,^4^. One generic challenge for algorithm design is to account for the effects of “outliers”, which often exist in clinical recordings due to not only external random influences but also intrinsic physiological/pathological incidence such as ectopic beats in ECG recordings^5^ (Fig. 1). For example, multiscale entropy analysis (MSE)^11^ is a useful tool for estimating the complexity of heartbeat fluctuations; and it can detect alterations in cardiac control with aging and predict clinical outcomes of patients with heart diseases^10^,^12^. However, MSE results are not reliable when heartbeat signals consist of outliers^13^,^14^. Thus, those data segments contaminated by outliers must be identified and excluded before performing MSE^14^. Such heavy-duty pre-processing is time consuming, thus compromising the clinical application of the analysis at the bedside. In addition, ignoring the segments with arrhythmia-related outliers may lead to loss of important information about the pathology of cardiac control. Therefore, there is an urgent need for the development of complexity analyses that can reliably quantify the degree of complexity in noisy physiological recordings with outliers.

In general, the change of a variable at a time point can be decomposed into two parts: the magnitude (absolute value) and the direction (sign)^15^. We hypothesize that dynamics in the sign time series can adequately reflect the complexity in raw data and that the complexity estimation based on the sign time series is more resilient to outliers as compared to raw data. Based on the hypothesis, we propose a new complexity analysis termed ‘multiscale symbolic entropy analysis’ (MSSE) that assesses the multiscale entropy of a signal from its sign time series. We also hypothesize that the new method can reliably detect pathological alterations of cardiac control based on the complexity of heartbeat fluctuations even when the signals are contaminated by ectopic beats. To test these hypotheses, we conducted numeric simulations and theoretical derivations to examine the performance of the new method for the analysis of signals with and without outliers. We also applied the method on human heartbeat recordings and examined whether complexity can be used to detect the alterations of cardiac control in patients with congestive heart failure (CHF) and in critically ill patients with certain dysfunctional organ(s) and life continuation relying on a mechanical circulatory support system, namely, extracorporeal membrane oxygenator (ECMO)^16^. Moreover, we compared the MSSE results with those of the traditional MSE.

## Results

### Assessment of complexity requires the examination of fluctuations at different time scales

The theoretical concept behind the MSE and MSSE as well as many other complexity analyses is that the complexity of a time series cannot be reliably determined by statistical properties such as fluctuation amplitude and entropy at a fixed time scale because these properties can vary with time scale^11^. To demonstrate this concept, we considered MSE results of (1) white noise that simply consists of uncorrelated data points, and (2) 1/f noise that is believed to represent the most complex fluctuation patterns in physical systems and is observed in many physiological systems under healthy conditions (see Methods). As shown in Fig. 2, the entropy value of a white noise can be smaller, equal to, or greater than that of a 1/f noise, depending on the time scale (and their standard deviations). Note that the entropy of a coarse-grained white noise at a time scale *l* is decreased by ln(*l*)/2 while the entropy of a coarse-grained 1/f noise remains approximately constant at all different times scales (Fig. 2 and Supplementary Material I). Thus, for the assessment of complexity, the entropy as the function of time scale (e.g., both the absolute values and the slope of the function) should be considered.

### MSSE provides the similar information as MSE for signals without outliers

To better account for the influence of outliers, we proposed a new algorithm, namely, multiscale symbolic entropy analysis, which quantifies entropies of fluctuations at different time scales (Fig. 3 and see details in Methods). For generated noise with different temporal correlations (Fig. 4 a to c), the two proposed MSSE measures (i.e., eSC and eEC) provided consistent results as the MSE measure does. For instance, eSC and eEC remained the same at different time scale for 1/f noise, decayed quickly at larger time scales for white noise, and decayed faster for anti-correlated noise. For noise with Hurst exponent >1 (stronger correlations as compare to 1/f noise), the entropy measures slightly increased with increasing time scales). Indeed both MSSE measures were highly correlated with the MSE measure at all time scales (Fig. 5). We further applied MSE and MSSE to heartbeat recordings of 26 healthy human individuals without outliers (Physionet: mean age: 31.7 ± 3.5 years old) (Red line in Fig. 6 a, g and j). Consistent with previous findings^13^, we found that all the entropy measures remained relatively constant at different time scales (except for very small time scales).

### MSSE is more resilient to outliers

To examine how outliers impact the performances of MSE and MSSE, we generated surrogate data by randomly replacing some data points in the normal heartbeat intervals of those healthy subjects with three different types of outliers that are due to the arrhythmic beats or QRS detection error (Fig. 7) (see details in Methods). The outliers significantly affected MSE, leading to overestimated entropies at time scales from 1–15 beats. The degree of overestimation depends on the time scales, i.e., more overestimation at smaller time scales. As a result, the MSE function became more like that of white noise (Fig. 6 a to c). Certain automatic filtering procedure^24^ has been proposed to address the time consuming procedure of manual filtering of outliers (Fig. 7, see details in Methods). Our simulation results showed that the automatic filtering could not suppress the effect of outliers on MSE (Fig. 6 d to f). This may be expected because many of simulated outliers were still present after the filtering (Fig. 7). In contrast, the results of MSSE remained virtually the same as those of the raw data, even when 45% artificial outliers were imposed (Fig. 6 g to l).

### Complexity reveals altered cardiac dynamics in diseases

**Figure 1:**
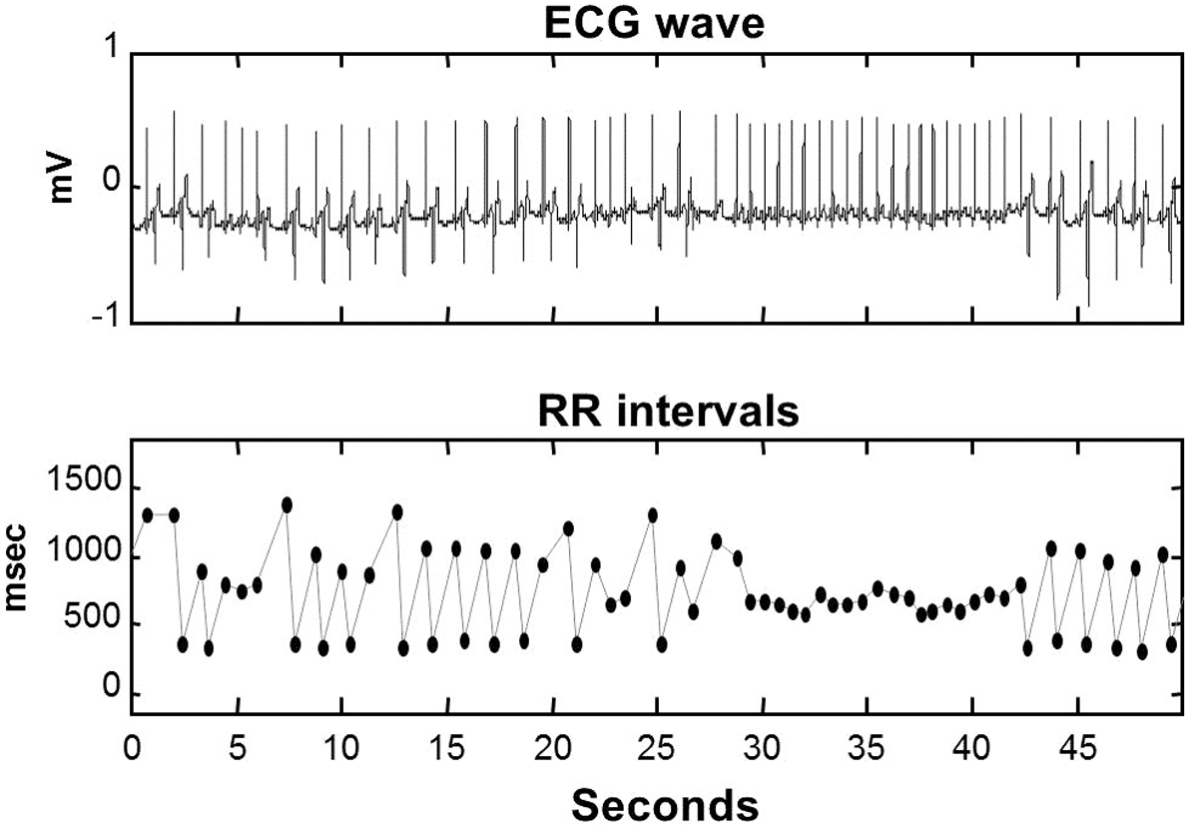
An ECG recording (upper) and derived heartbeat intervals (lower) of a critical ill patient using ECMO. The frequent arrhythmias in the recording lead to many outliers in the derived heartbeat signal.

We next applied the MSSE and MSE to human heartbeat recordings of four additional older groups (see Methods): (1) 46 older control subjects; (2) 29 patients with congestive heart failure (CHF) who still maintained daily activities; (3) 33 critical ill patients who were hospitalized with the support of extracorporeal membrane oxygenator (ECMO) and eventually survived, and (4) 31 ECMO patients who died. We selected the four older groups because (i) their clinical health conditions are clearly distinguishable (i.e., as compared to the controls, health is reduced in CHF patients, further reduced in ECMO patients, and mostly reduced in ECMO patients who did not survived), and (ii) ectopic beats are very common in these subjects (Fig. 1), especially in ECMO patients. Thus, without filtering the ectopic beats, the data of the four groups allowed us to test whether or not the introduced complexity analysis as well as the traditional MSE can enable a reliable assessment of complexity in RR time series with outliers and detect the alterations in cardiac dynamic under different health conditions.

Overall there were significant group differences in all entropy measures. The differences between the control and CHF subjects were present exclusively at small time scales (<~5 heartbeats). Specifically, the mean eEC at <~5 beats showed a significant difference between the two groups (i.e., the CHF group has smaller eEC); and the slopes of MSE and eEC functions at <~5 beats were consistently reduced in the CHF patients as compared to the controls (Table 1). In addition, the dependences of entropy measures on time scales in CHF patients behaved (Fig. 8) more like that of correlated signals with H > 1 (Fig. 4 b to c), e.g., the slope of eEC at scales 2–10 (0.084 ± 0.10) was larger than that of the older controls (0.048 ± 0.063, p < 0.05). These results are consistent with the MSE results as reported previously^10^,^11^,^13^, indicating reduced complexity in heartbeat fluctuations in CHF patients.

As compared to the older controls, the ECMO patients had much lower values of MSSE measures at all time scales (p < 0.0001), suggesting significantly reduced heartbeat variability (see Table 1 and Fig. 8). Similar to the older controls, the ECMO patients also displayed a crossover in the MSSE functions (e.g., see the profile of eEC in Fig. 8). However, unlikely the controls, the slope of eEC function at time scales below the crossover was negative in ECMO patients, resembling those observed in white noise or anti-correlated noise (Figs. 4b–c). These results suggest altered/disrupted cardiac control in these ECMO patients. Moreover, the changes of MSSE results in the ECMO group (i.e., reduced entropy values at all time scales and reduced slope at small time scales) were much more pronounced in those patients who died as compared to those who survived (see Table 1), suggesting more degraded cardiac control in the ECMO patients with fatal outcomes. At time scales >5 beats, eEC of ECMO survivors slightly increased with time scales (Fig. 8), suggesting a behavior similar to fractional Brownian noise with Hurst exponent >1.

Consistent with the MSSE results, the MSE function of ECMO patients also showed a negative slope at small time scales. But the slope was not different between the survived and the deceased patients (Table 1). The most unexpected results were that the MSE-derived entropy values of the ECMO patients, especially the survived patients, were close to or even larger than those of the older controls at all time scales (see Table 1). This discrepancy is likely due to arrhythmia-related outliers in these signals that can significantly affect the performance of MSE, leading to artificial increases in entropy values as shown in the simulations (Fig. 6 a). Thus, the results of ECMO data provide further evidence for the limitation of MSE.

It should be noted that MSSE as well as the traditional MSE have the issue of threshold effect (see the details in Supplementary III). The variation between two normal heartbeat intervals in the critically ill patients usually becomes very small, likely as a consequence of reduced autonomic nervous activity. Thus, the difference between two consecutive data points in the coarse-grained time series at large scales hardly exceeds the quantization error such that the sign series contain mainly zeros. Consequently, entropy measures are expected to become relatively stable at very large time scales, which was observed at scales >7 beats in ECMO patients (Fig. S3).

## Discussion

With the emergence of the interdisciplinary field of nonlinear dynamics in medicine, how to extract health-related information in ECG-derived heartbeat fluctuations has attracted more and more attentions. It is hypothesized that complexity in heartbeat fluctuations reflects healthy cardiac control and reduced complexity in the fluctuations indicates degraded cardiac control as occurred with aging and under pathological conditions^10^,^12^,^13^,^15^. Our results confirm this hypothesis and further show that cardiac complexity can predict survival of the critically ill patients who used ECMO to sustain their lives.

**Figure 2:**
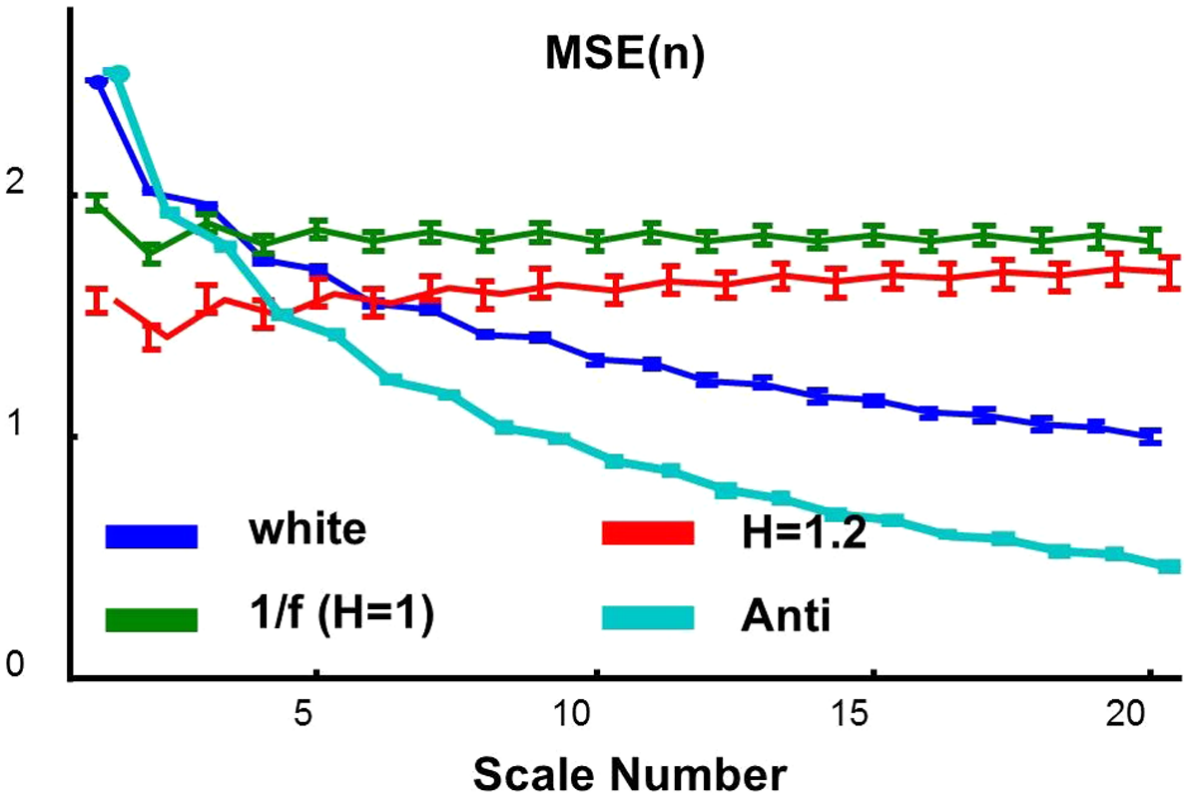
Sample entropies of surrogate data with Hurst exponent (H = 0.4–1.2) at different time scales.

Complex heartbeat fluctuations is believed to stem from the interconnectedness of physiological mechanisms that is facilitated by a network of control nodes with feedback interactions^1^. Such complexity is manifested by many nonlinear features, including strong correlations at multiple time scales^17^,^18^ that can be assessed by fractal analysis such as detrended fluctuation analysis (DFA)^19^,^20^,^21^. Based on the estimation of randomness, multiscale entropy analyses such as MSE and MSSE also can be used to determine multiscale correlations by examining the relationship between entropy and time scale (Figs. 2, 4). For example, a negative slope of the entropy function indicates anti-correlated (i.e., simple oscillation, a repetitive pattern of an increase follow by a decrease) or uncorrelated fluctuations with the loss of feedback interactions^13^. Thus, the negative slope in the entropy function of the deceased ECMO patients suggests significantly reduced correlations in heartbeat fluctuations that are expectedly caused by the loss of feedback interactions in cardiac control of these patients. This finding provides evidence that reduced heartbeat correlations could predict the outcome of severely ill patients.

The proposed complexity analysis is based on the examination of the sign time series of a signal and its coarse-grained signals at different time scales. As we showed in our simulations, this approach can minimize the effect of randomly distributed outliers, thus helping to reveal the true dynamics in signals. Regarding the approach and simulations, there are a few points worth clarifying. First we note that ectopic beats do not necessarily occur randomly^22^. Thus, it is likely that the temporal distribution of ectopic beats in a real heartbeat signal may reflect certain aspects of cardiac control and/or pathological changes. More systemic studies are required to test how those ectopic beats contribute to complexity in heartbeat fluctuations. Second, by focusing on sign series, we do not imply that the magnitudes of a signal contain no useful information. Indeed the magnitude time series of a signal may contain dynamic information that is complementary to that in the sign series^15^. We sacrificed the possible useful information in the magnitude series because it can be easily contaminated by outliers. Finally, in MSSE, we proposed to use two entropy measures (i.e., eEC and eSC) to estimate the irregularity of the sign series at each time scale. Actually the MSSE results appear to be not sensitive to the method of estimating the irregularity, and similar results can be obtained using an alternative approach for the estimation of entropy in sign time series (see Supplementary Material II).

The number and severity of critically ill patients increase worldwide such that it is crucial for critical care professionals to make prudent and objective decisions on the allocation or termination of costly and risky treatments such as ECMO for these patients. Currently, only about half of the adult patients receive ECMO^23^. Due to the high cost of the treatment, it is important to identify patients who are likely to benefit from ECMO and to determine the appropriate timing of stopping ECMO. Physiology-based risk-classification tools are therefore needed to support decisions for or against continuous ECMO usage. Monitoring ECG is a routine procedure in clinical setting. Successfully applying the research findings of complexity in heartbeats to clinical practice (e.g., the use of ECMO) will have huge impacts on healthcare and medicine (e.g., ECMO usage). However, such a potentially important application has been impeded by the fact that, exclusively all previous complexity analyses require heartbeat signals without outliers or ectopic beats. This requirement is important because outliers can change significantly the estimated complexity based on the traditional complexity analyses such as MSE (Fig. 6a).

Removing outliers or ectopic beats is not trivial, not only requiring specific expertise in ECG waveforms but also being very time-consuming. The recursive automatic filtering proposed by Molina–Picó *et al.* has been shown to be able to reduce the effect of outliers on some heart rate variability measures^24^. However, the automatic filtering only slightly improved the MSE results while the effect of outliers on MSE mostly remained (see Fig. 6 d to f). Moreover, it is worth noting that the threshold-based filtering only works in the signals with the occasional and isolated ectopic beats but may not be applicable in the data with numerous and often continuous ectopic beats as occurred in ECG data of ECMO patients (see lower panel of Fig. 7). The proposed MSSE was specially designed to resolve these problems. With its reliability and high resilience to outliers, the method raises the possibility of applying the theory of complexity in clinical practice. Further validation of the method using a large sample size is warranted.

## Methods

### Multiscale entropy analysis

As described previously^11^, MSE calculates the degree of irregularity in the fluctuations of a signal, {*X_i_*}, at different time scales *l*. For each time scale, the time series is first coarse-grained to produce a new time series 
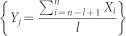
, where *n* = *l* × *j*. Then the degree of irregularity in {*Y_j_*} is estimated using sample entropy (SpEn).

### Multiscale symbolic entropy analysis

**Figure 3:**
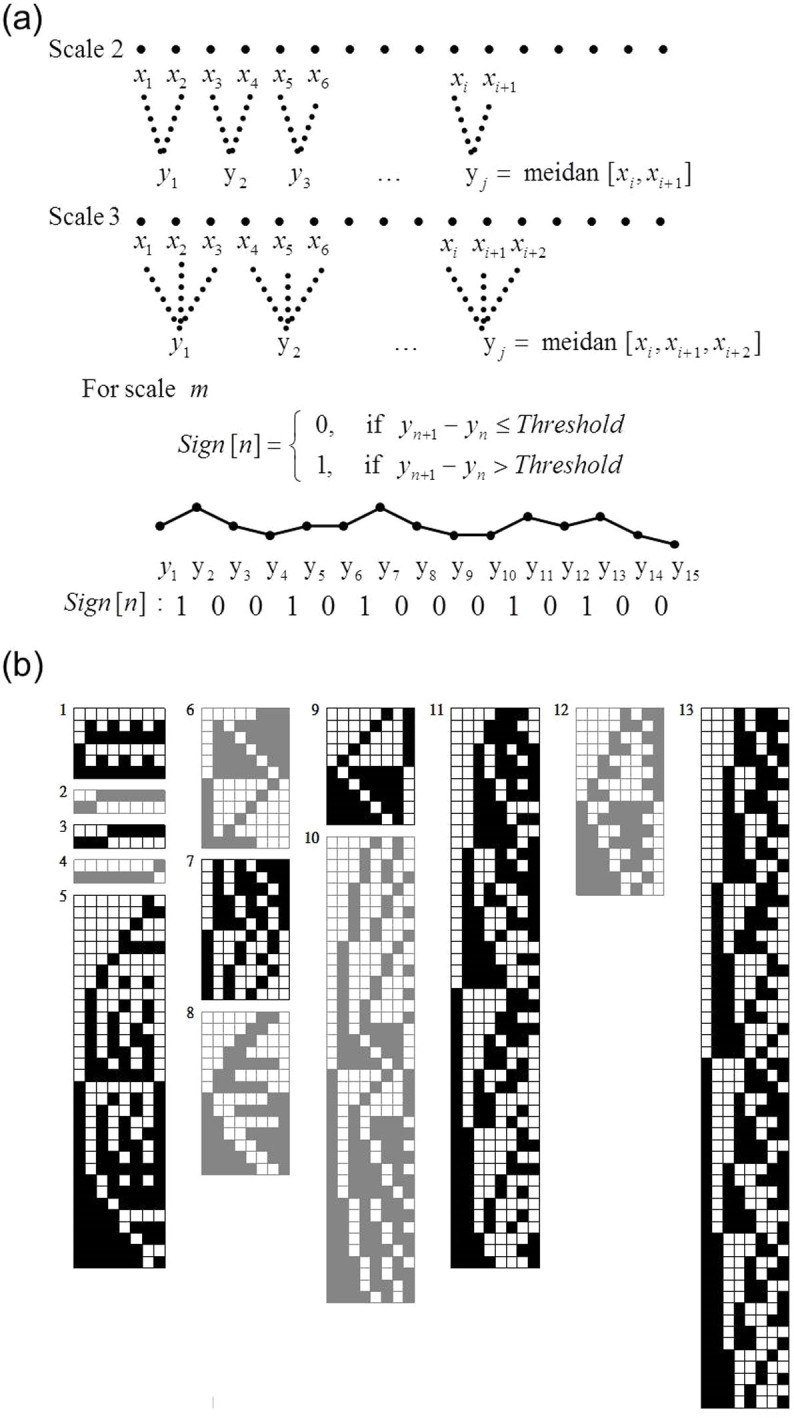
Diagram of the procedure for the multiscale symbolic entropy analysis. (a) To coarse grain a signal at a chosen scale (e.g., the cases for scale = 2 and 3 are shown in the top two panels, respectively), the original data series is first divided into non-overlapped boxes with the size of the scale. Then the median of all points in each box is calculated. The sign series of a coarse grained signal is generated by considering the direction of change at each point (i.e., 1 for increasing and 0 otherwise). (b) The sign series is divided into *m*-bit sequences that are then categorized based on their patterns. To avoid assigning sequences with similar patterns to different categories, *m*-bit sequences are categorized and ranked according to their conditional probabilities. In this example, *m* = 8 and there are 13 possible categories. Finally, based on the distribution of the sequences in different categories, the Shannon entropy eSC and the mean rank (namely, symbolic sample entropy) eEC are obtained for the sign time series.

**Figure 4:**
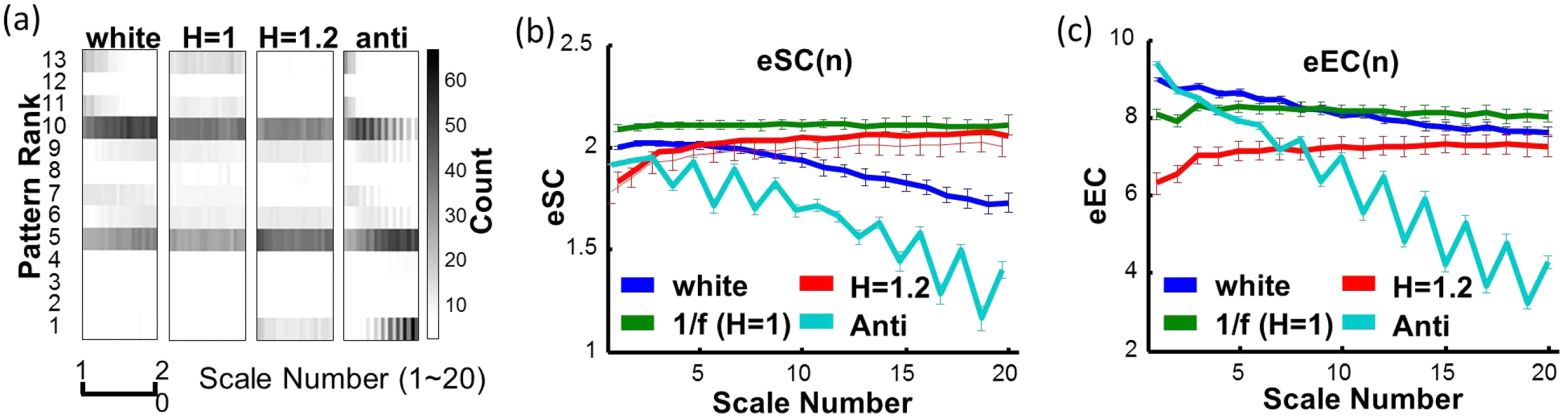
MSSE results of generated noise and human heartbeat fluctuations. (a) The probability distributions of 8-bit sequences for noise with different correlations as characterized by Hurst exponent (H) = 0.4, 0.5, 1.0, and 1.2, respectively. For all signals, data length = 30,000 points and *m* = 8-bit. There are 13 possible categories as shown in Fig. 3b. The gray scales indicate the probabilities in these categories. For white noise, more and more 8-bit sequences follow certain patterns as time scale increases; and 1/f signal has basically the same probability distribution of sequence patterns at different time scales. (b–c) MSSE results of generated noise used in (a). Both proposed MSSE measures as well as the MSE (Fig. 2) remain virtually unchanged at different time scale for 1/f noise, and decay fast as time scale increases for H < 1. For H > 1, the two different symbolic entropies slightly increase with time scales but stay below the values of 1/f noise.

**Figure 5:**
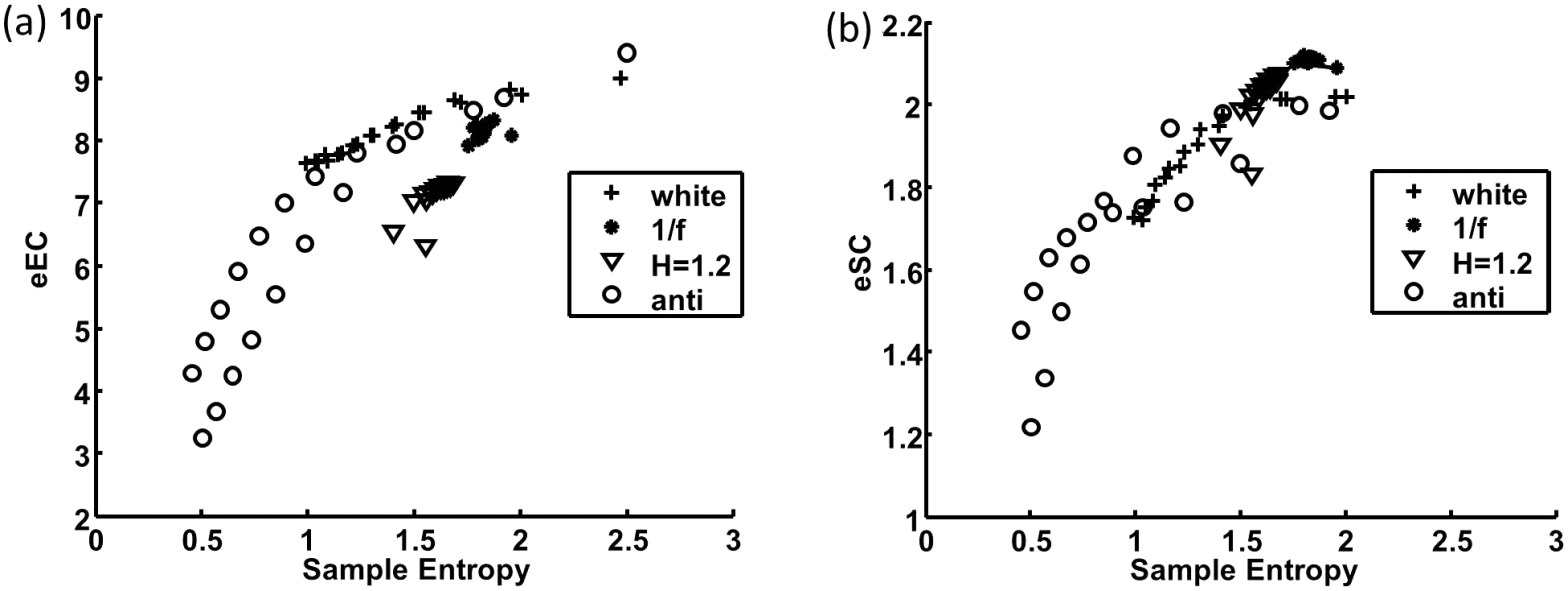
MSSE measures are correlated with the MSE measure. The scatter plots of (a) sample entropies vs eEC and (b) sample entropies vs eSC at different time scales (1–20) for signals with different Hurst exponent (H = 0.4–1.2).

**Figure 6:**
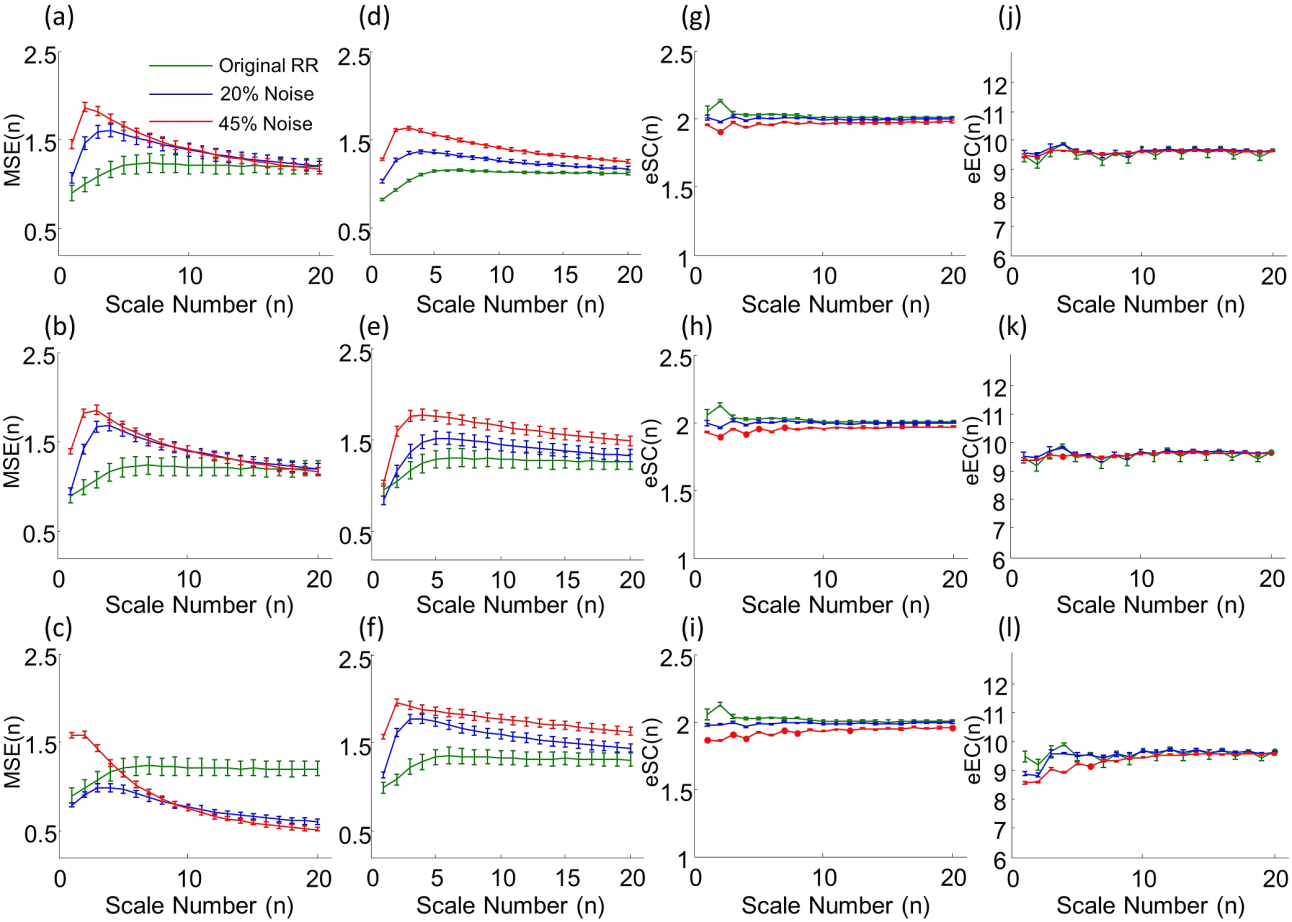
Influences of outliers on MSE and MSSE. (a–c) MSE of heartbeat recordings of 26 healthy young subjects and the corresponding surrogate data with different types of outliers: (a) arrhythmic beats selected based on arrhythmia heartbeat intervals of 29 patients with CHF (Congestive heart failure RR interval database in Physionet), (b) simulated premature ectopic beats, and (c) spurious peaks due to R wave detection errors. (d–f) MSE results after the recursive autonomic filtering (iteration number is 5). (g–i) eEC and (j–l) eSC of heartbeat signals and surrogate data that are used in (a–c). Results are shown as group mean ± standard error.

To better account for the influence of outliers, we propose a new algorithm, namely MultiScale Symbolic Entropy (MSSE) analysis. Different from MSE, MSSE considers the sign time series 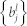 of each coarse-grained series at a time scale *l*. (Fig. 3a), where 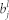 is either 1 when the corresponding 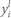 is increasing or 0 otherwise. To consider the quantization error in digital signals (e.g., 4 msec for signals with a sampling rate of 250 Hz), let 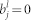 if the amplitude of a change is less than the quantization error. In addition, median values rather than mean values in non-overlapped windows are used to construct coarse-grained time series in order to minimize the impact of outliers (Fig. S4).

To quantify the irregularity of a sign time series, the signal is first divided into sequences each with the same length *m* — the sequence length that is pre-selected (by default, *m* = 8 in this study). These *m*-bit sequences are divided into different categories based on their temporal patterns using the similar concept of the approximate/sample entropy^25^. Specifically, an *m*-bit sequence is divided into multiple vectors, each consisting of *D* consecutive bits {(*b*_1_, *b*_2_…*b_D_*);(*b*_2_, *b*_3_…*b_D_*_+1_);…}, where *D* is the dimension of vectors. The number of paired vectors consisting of the exactly same binary codes is then obtained and is denoted as *n*(*D*). The conditional probability of the sequence is determined by *n*(*D* + 1)/*n*(*D*). All sequences are assigned to different categories based on their conditional probabilities (i.e., sequences in a category have the same conditional probability). Categories are created using all possible *m*-bit sequences (not only the sequences present in a sign time series) and ranked based on the conditional probability, i.e., the conditional probability is the highest for the category with Rank = 1 and lower for the categories with lower ranks (Fig. 3). Then, based on the distribution of the sequences in different categories, the Shannon entropy eSC(*l*) and the mean rank (namely, symbolic sample entropy) eEC(*l*) can be obtained for the sign time series. Conceptually, eSC describes the information richness of a signal while eEC indicates the degree of uncertainty of the fluctuations.

### Human heartbeat recordings

To test the performances of complexity analyses, we utilized existing heartbeat recordings of three groups of human subjects: (1) 26 healthy young subjects without outliers at age of 31.7 ± 3.5 (SE) years old; (2) 46 older control subjects at age of 65.9 ± 4.0 (SE) years old (24 hours); (3) 29 patients with congestive heart failure (CHF) at age of 55.2 ± 11.6 (SE) years old (24 hours); and (4) 64 critically ill patients at age of 53.5 ± 18.2 (SE) years old who had severe dysfunction in certain organ(s) (i.e., fulminant myocarditis, severe respiratory failure, cardiogenic shock after cardiac surgery and septic shock)^16^,^26^,^27^ and relied on the extracorporeal membrane oxygenator (ECMO) to maintain life continuation (24 hours). Within the 64 ECMO patients, 33 survived while the others died.

The data of the first three groups are from the existing databases that are publically available in *Physionet.org*^18^. The data of Group 4 were collected in the National Taiwan University (NTU) Hospital between March 2008 and March 2010. Patients were eligible for the present study if they were 18 years or older and had received ECMO for circulatory or respiratory failure that required mechanical support. The decision to use ECMO was made by experienced intensive care specialists or cardiac surgeons. The primary endpoint is death or urgent cardiac transplantation during the index admission. The patients were followed until discharge or death of the index admission. The Institutional Review Board of the NTUH approved the study and informed consent was obtained from each patient's next-of-kin in ECMO group and from each subject in control group in accordance with the NTU's human subject's research polices.

### Surrogate data

We generated noise with different correlation properties by using a modified Fourier filtering method^28^. The generated signals possess the desired power-law correlation functions that asymptotically behave as fractional Brownian motion (fBM) processes with different Hurst exponent (H) (see Supplementary I): white noise (Hurst exponent = 0.5), 1/f noise (Hurst exponent = 1), noise with stronger correlations (Hurst exponent = 1.2), and signals with anticorrelations (Hurst exponent = 0.4). For each type of noise, we generated 1000 signals each with 30,000 points.

**Figure 7:**
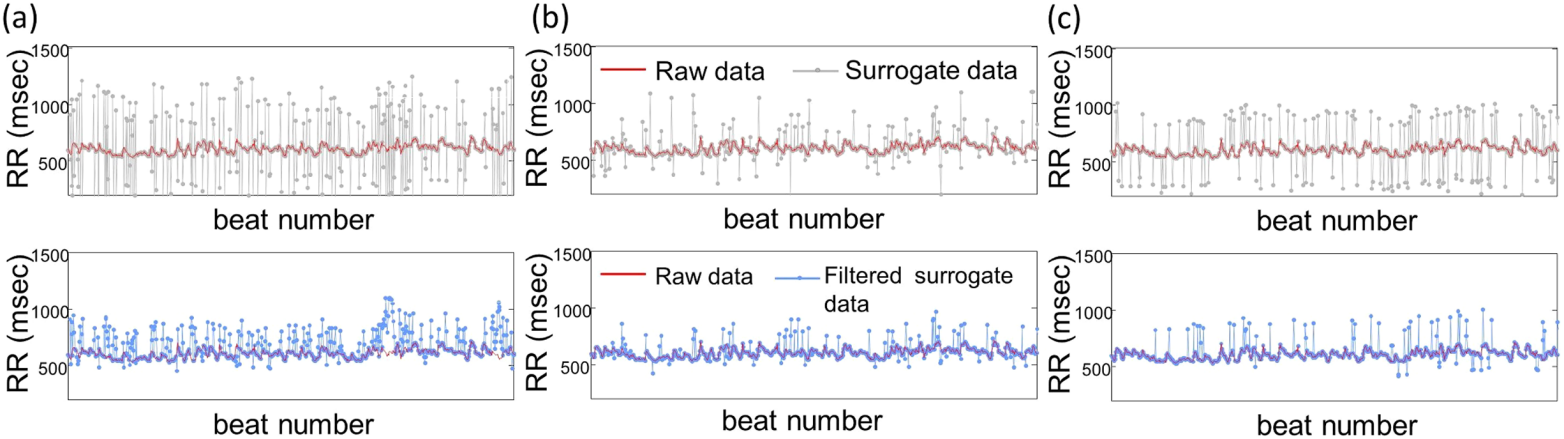
An RR series of a healthy young subject (red) and surrogate data from the RR series (dotted grey lines). Surrogate data were generated by randomly replacing 20% of data points in the RR series with different artificial outliers which are (a) simulating premature ectopic beats, (b) arrhythmic beats selected based on arrhythmia heartbeat intervals of the patients with CHF in Physionet database and (c) spurious peaks due to R wave detection errors. The surrogate data after autonomic filtering (see Methods) are provided in the lower panels.

**Figure 8:**
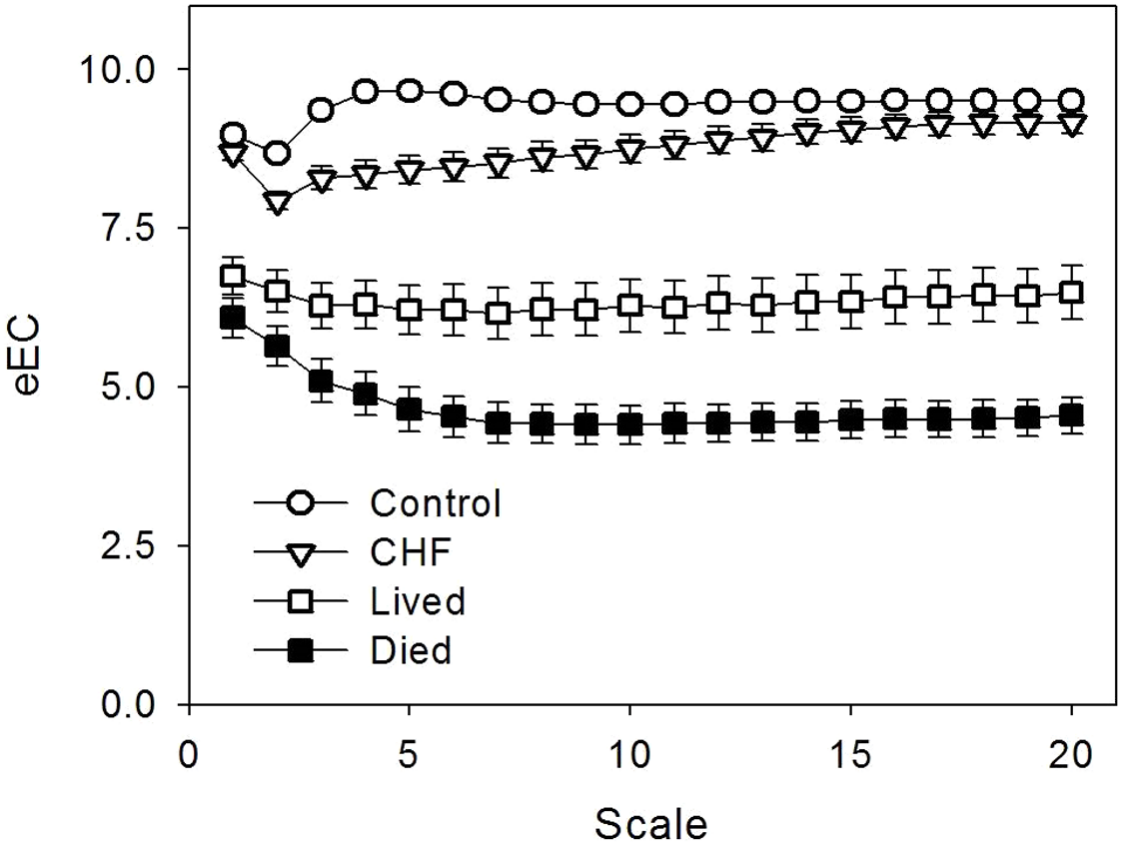
MSSE results of heartbeat recordings collected from older controls, CHF patients, and ECMO patients (survived and expired). Results were obtained without ectopic beat rejections. Data are shown as group averages and error bars indicate standard errors.

Human heartbeat recordings with artificial ectopic beats are generated from total 24-hour heartbeat signals collected from 26 healthy young individuals. The signals have been previously reviewed and contain no abnormal beats (www.physionet.org\…). For each recording, three different ways were adopted to simulate the ectopic beats. We randomly selected certain percentage (e.g., 20% and 45%) of RR intervals. Then we replaced the normal beats with either (1) intervals imitating premature ectopic beats, i.e., each simulated ectopic beat leads to an abnormal RR interval that is ~65 percent (30%–100% in uniform distribution) of the mean value of the four normal hear beat intervals proceeding to the ectopic beat^29^ (Fig. 7 a); or (2) artificial outliers that are selected based on arrhythmia heartbeat intervals of the patients with CHF in Physionet database (Fig. 7 b). In addition, we also consider the influence of the spike train that simulates the spurious peaks due to R wave detection errors^24^. The spike trains were generated using a Bernoulli process of probability p (e.g., 0.2 and 0.45): 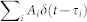 where *A_i_* is the spike amplitude and *τ_i_* is the temporal location of the spike. The positive and negative spikes indicate the missing and false positive heart beat detection, receptively. In this study, the amplitude of each spike was assigned to be a value randomly selected from a normal distribution with mean equal to half the median of 10 adjacent RR intervals and standard deviation equal to that of the same 10 referencing beats.

### Autonomic filter

Certain automatic filtering procedure has been proposed in previous studies to address the time consuming procedure of manual filtering. For example, Molina–Picó *et al*. proposed a recursive filtering procedure^24^ in which a signal is scanned with a sliding window of the size = 10 beats and an RR interval is identified as an outlier if its value significantly deviates from the mean value of its nearby points (i.e., 5 beats preceding and 5 beats following the point). The degree of deviation is a predetermined threshold, e.g., (25% and 50% of the mean for upper and lower bounds respectively). For an identified outlier, the RR value is replaced by the linear interpolation of two adjacent beats (see Fig. 7). It is necessary to scan the time series multiple times (i.e., iteration number > 1) in order to identify certain clustered outliers^29^.

## Author Contributions

M.T.L., Y.L.H., C.K.P. and K.H. conceived and designed the research. Y.H.L. and Y.L.H. carried out the experiment. Y.C.C., H.W.V.Y. and M.T.L. performed the data analysis. C.L. performed the statistical analysis. All authors wrote the manuscript.

## Supplementary Material

Supplementary Information Supplementary Documents

## Figures and Tables

**Table 1:** Parameters derived from different entropy analyses of short- and long-time scales

	Summation of Scale 1–5					Summation of Scale 6–20					Slope (scale 2 to 5)				
Variable	Ctrl	CHF	Survival	Expired	P	Ctrl	CHF	Survival	Expired	P	Ctrl	CHF	Survival	Expired	P
MSE	1.5 ± 1.7	0.86 ± 1.1	2.8 ± 2.5*^†^	1.7 ± 1.6	<10^−3^	5.3 ± 5.7	3.1 ± 4.5	7.7 ± 5.8†	3.6 ± 3.5#	<10^−2^	.06 ± 0.04	.02 ± 0.05*	−.02 ± .06*†	−.03 ± .05*†	<10^−12^
eSC	10.7 ± 0.2	10.6 ± 0.5	9.2 ± 1.5*†	8.2 ± 1.7*†#	<10^−15^	31.2 ± 0.6	31.9 ± 1.2	27.1 ± 5.8*†	23.3 ± 5.7*†#	<10^−15^	−.01 ± 0.02	−.01 ± 0.03	−.02 ± .06	−.08 ± .04*†#	<10^−11^
eEC	46.4 ± 1.3	41.7 ± 4.6*	32.1 ± 9.8*†	26.4 ± 9*†#	<10^−15^	142.5 ± 2.6	133.5 ± 15	95.0 ± 35.9*†	67.3 ± 24.7*†#	<10^−15^	.33 ± .15	.15 ± 0.24*	−.09 ± .29*†	−.32 ± .18*†#	<10^−15^

All data are expressed as mean ± S.D. Analysis of Variance of those entropy indexes is performed. For the cases with significant group differences (P < 0.05), post hoc tests with Bonferroni correction are then used for the comparisons between different pairs of two groups:

*indicating p < 0.05 for the comparison with controls;

†for the comparison with CHF patients; and ^#^ for the comparison with the survival ECMO patients.
