# Supplementary material for: Outlier-resilient complexity analysis of heartbeat dynamics
Source: Sci Rep. 2015 Mar 6;5:8836. doi: 10.1038/srep08836 (PMC4351527; doi:10.1038/srep08836)
Supplement: Supplementary Information — Supplementary Documents [file srep08836-s1.pdf]

Supplementary Information

Outlier-resilient complexity analysis of heartbeat dynamics

Men-Tzung Lo[1,2], Yi-Chung Chang[1,3] , Chen Lin[1] , Hsu-Wen Vincent Young[1],Yen-Hung Lin[4],

Yi-Lwun Ho[4*], Chung-Kang Peng[5] and Kun Hu[2,6*]

[1]*Research Center for Adaptive Data Analysis & Center for Dynamical Biomarkers and Translational Medicicne, National Central University, Taoyuan, Taiwan*

[2]*Medical Biodynamics Program, Division of Sleep and Circadian Disorders, Brigham and Women's Hospital, 221 Longwood Avenue, Boston, MA 02115, USA*

[3]*Graduate Institute of Communication Engineering, National Taiwan University, Taipei , Taiwan*

[4]*Department of Internal Medicine, National Taiwan University Hospital and National Taiwan University College of Medicine, Taipei, Taiwan*

[5]*Division of Interdisciplinary Medicine and Biotechnology, Beth Israel Deaconess Medical Center/Harvard Medical School, Boston, Massachusetts, USA*

[6] *Division of Sleep Medicine, Harvard Medical School, Boston, MA, USA*

* *Correspondence to be sent to:*

Division of Cardiology, Department of Internal Medicine, National Taiwan University Hospital, 7 Chung-Shan South Road, Taipei, Taiwan.

Email: ylho@ntu.edu.tw

or

Medical Biodynamics Program, Division of Sleep and Circadian Disorders, Brigham and Women's Hospital, Harvard Medical School, Boston, MA 02115, United States;

Email: khu@bics.bwh.harvard.edu

Supplementary I

The differential entropy of a given probability density function, $\{p(x) \mid x \in X\}$, is defined as:

$S_p(\mathrm{X}) = \int_X -p(x)\ln(p(x))dx$, which is a generalized Shannon entropy in the case of continuous probability distribution. For a Gaussian (normal) distribution,

$$p(x) = \frac{1}{\sqrt{2\pi\sigma^2}}\exp\left(-\frac{(x-\mu)^2}{2\sigma^2}\right) \;,\; S_P(X) = \ln\left(\sigma\sqrt{2\pi e}\right).$$

Noise with different correlations, $x(t)$, can be generated by the fractional Brownian motion (fBM) processes with different Hurst exponent (H). The correlation function can be expressed as $\langle x(t), x(s)\rangle = \frac{1}{2}\left(t^{2H'} + s^{2H'} - |t-s|^{2H'}\right)$, where t and s are greater than zero (it can usually assumed that the process starts at time zero) and $H' = H - 1$. Though an fBM is not necessarily stationary, an averaged spectrum can still be defined for such processes, i.e., the spectrum $S(\omega)$ is proportional to an inverse power of ω, namely, $S(\omega) \approx \frac{1}{|\omega|^{2H'+1}}$ (see [1]).

1/f noise can be viewed as the fBM with H'=0 (H=1), and can be treated as a stationary process. Based on the equations above, the correlation function and the variances) of 1/f noise are constant and independent of time scale. Thus, coarse graining the process changes nothing about the correlations for the 1/f noise (since it is just multiplying a certain number by l^2 and then dividing it by l^2), and the structure of the coarse-grained 1/f series remains the same. Therefore, the entropy should also remain approximately constant. We note that there are certain limitations in the above derivation, especially for real signals. For instance, the spectrum shown above is not integrable and the stated mathematical relations are not always rigorously derived. In addition, in order for the process to be physically meaningful, we must have both a lower and upper cutoff frequencies for the spectrum of 1/f noise. Thus,t he correlation functions are only approximately constant for a certain range of time scales. Nevertheless, for the time scales and the signals that are interest for most of studies, all the mathematical relations mentioned above still hold essentially, as

confirmed by various numerical and experimental results.

[1] P. Flandrin, Information Theory, IEEE Trans **38.2,** 910 (1992).

Supplementary II

In order to avoid assigning similar patterns of sequences (they might carry the same information in different disguises, or be transformed into each other in an exact way) to different categories, a set of rules is used to identify related patterns. For instance, a binary sequence can be doubled flipped, or slided (the circular ring concept). All the possible sequences that can be generated from an *m*-bit sequence via these transformations and their combinations should be identified and assigned to the same category as the original sequence (Figure S1). Shannon entropy measured from the relocated patterns (m -bits sequence, m =6) and corresponding probability is denoted as eSF(l). Figure S2 shows the results of simulations for the white noise and the 1/f noise.

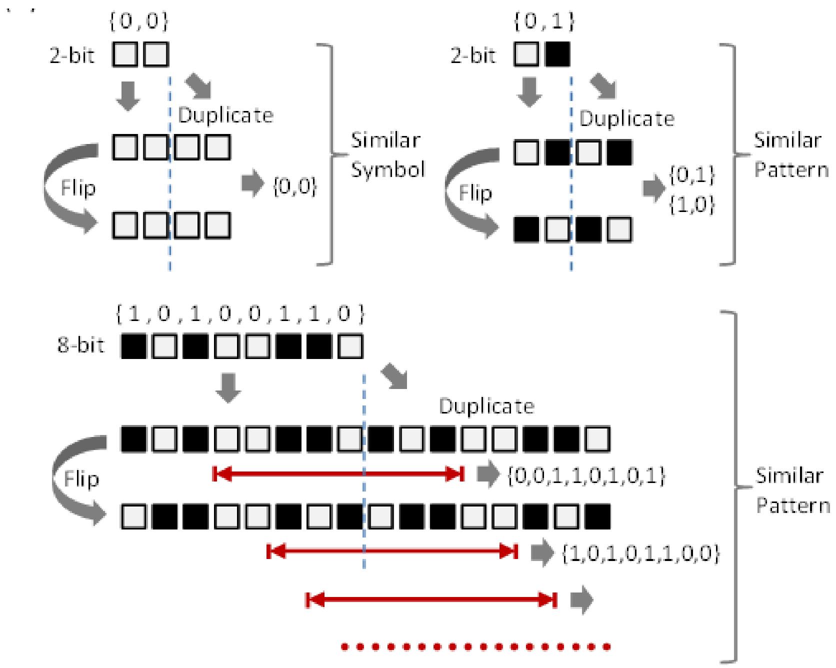

Figure S1: Related sequence patterns. Given an n-bit binary sequence, it can doubled after being flipped or inverted (meaning 1-> 0, 0->1) or left unchanged. An n-bit window can also slide through the transformed and doubled sequence.

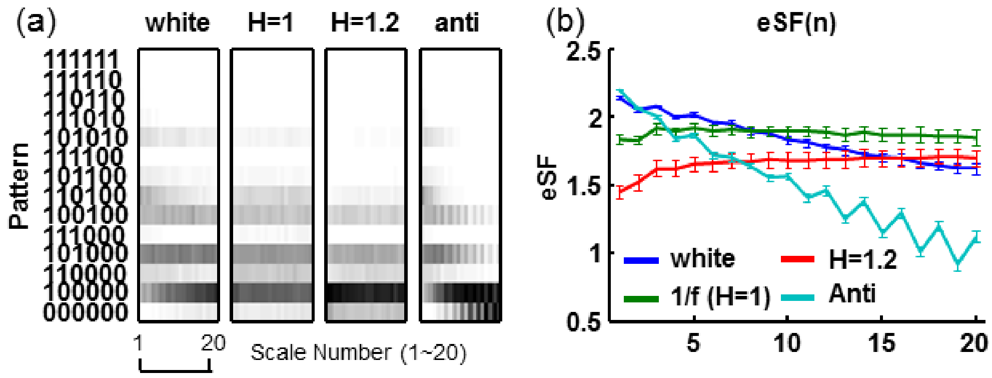

Figure S2: Shannon entropy of sign series of noise with different correlations. (a) Probability distribution of 6-bit sequences in different pattern categories. The length of all time series is 30000 points. The gray scales indicate the probabilities in different categories. For white noise, more and more sequences display the same patterns that belong to certain categories (i.e., the distribution becomes narrower) when the time scale increases; the more complex 1/f signal has virtually the same probability distribution for all patterns at all time scales. (b) Shannon entropy as functions of time scale. For all noise with different correlations, the eSF functions show the similar, consistent behaviors as eEC and eSC, i.e., white noise and anticorrelated noise with entropy decaying fast with increased scales for H <1; 1/f noise (H=1) with stable entropy values at different time scales; and more correlated noise (H>1) with slightly increased entropy at larger time scales.

Supplementary III

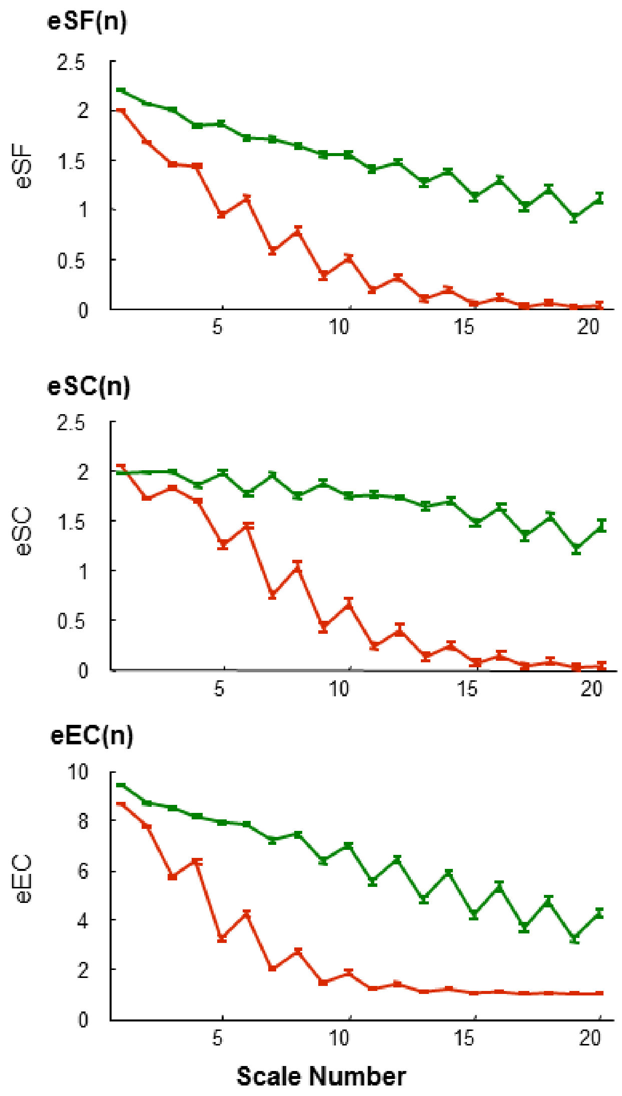

Figure S3: Effects of quantization on entropy measures at different time scales. Using the same quantization level, three different entropy measures as functions of time scale are obtained from the random signals (green) and the same time series but with artificially reduced amplitude reduced by half (red). The mean value and standard deviation are derived from 100 realizations of surrogate data. For the signals with reduced amplitude, all three symbolic entropy measures decay faster at small scales (as compared to original signals). In contrast, at very large time scales (>15), the effect of quantization leads to a saturation behavior, i.e., the entropy value function of the noise converges to a constant value.

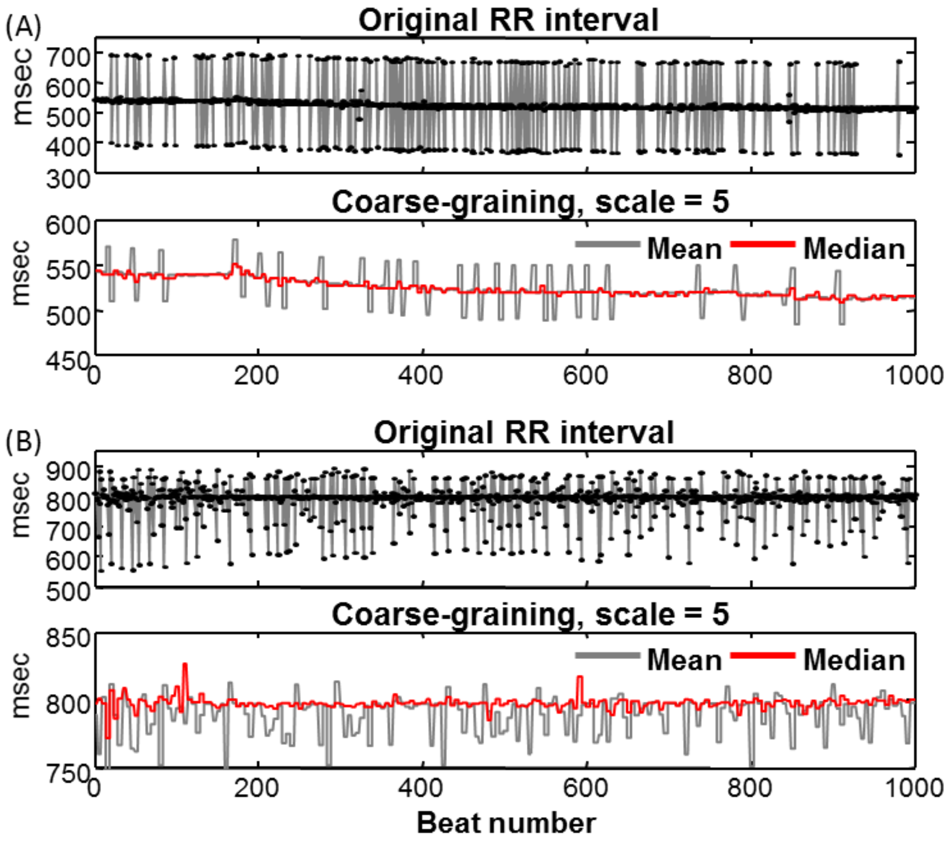

Figure S4: Two examples of RR series collected from the patients using ECMO. The bottom panel shows the time series coarse grained with a scale factor of 5 using median values (red) and mean values (gray). The two methods of coarse graining provide similar results except that using median values results in less abrupt spikes that are caused by arrhythmia-related outliers.
